# Size-Dictionary Interpolation for Robot’s Adjustment

**DOI:** 10.3389/fbioe.2015.00063

**Published:** 2015-05-19

**Authors:** Morteza Daneshmand, Alvo Aabloo, Gholamreza Anbarjafari

**Affiliations:** ^1^Intelligent Materials and Systems (IMS) Laboratory, Institute of Technology, University of Tartu, Tartu, Estonia; ^2^Intelligent Computer Vision (iCV) Group, Institute of Technology, University of Tartu, Tartu, Estonia

**Keywords:** classification according to gender and size, size-dictionary interpolation, 3D human body scanning, body-shape-changing mannequin robots, biomimetics, biologically inspired actuators

## Abstract

This paper describes the classification and size-dictionary interpolation of the three-dimensional data obtained by a laser scanner to be used in a realistic virtual fitting room, where automatic activation of the chosen mannequin robot, while several mannequin robots of different genders and sizes are simultaneously connected to the same computer, is also considered to make it mimic the body shapes and sizes instantly. The classification process consists of two layers, dealing, respectively, with gender and size. The interpolation procedure tries to find out which set of the positions of the biologically inspired actuators for activation of the mannequin robots could lead to the closest possible resemblance of the shape of the body of the person having been scanned, through linearly mapping the distances between the subsequent size-templates and the corresponding position set of the bioengineered actuators, and subsequently, calculating the control measures that could maintain the same distance proportions, where minimizing the Euclidean distance between the size-dictionary template vectors and that of the desired body sizes determines the mathematical description. In this research work, the experimental results of the implementation of the proposed method on Fits.me’s mannequin robots are visually illustrated, and explanation of the remaining steps toward completion of the whole realistic online fitting package is provided.

## 1 Introduction

Given the time consumption caused by the actual try-on of lots of clothes to decide on the best possible one in the sense of texture, material, color, size, etc., a virtual alternative has always been desired, and attracted the attention of so many scientists from different areas during the few last decades (Holte, 2013; Adnan et al., 2014; Gao et al., 2014). The foregoing procedure, ideally, should be implemented via realistic 3D modeling of body and garment. For example, making use of iterated graph cuts for extracting the background has been suggested in Rother et al. (2004), and factorized graph matching has been investigated in Zhou and De la Torre (2012). However, the latter list of attempts has not been successfully achieved their goals yet, although being paid serious attention by numerous researchers (Protopsaltou et al., 2002; Zhang et al., 2009; Sengupta and Chaudhuri, 2013).

This paper conducts research supporting creative industries fostering production of web-based realistic reverse simulation-based 3D virtual fitting room, and explores the potential of 3D technology to enhance the idea of fitting outfits through Internet without actually wearing them.

Recently, many researchers have started to work on developing virtual systems, which are dealing with clothing and fashion industry (Chen et al., 2012; Li and Li, 2012). A commercially applicable virtual fitting room could significantly grow the online clothing market. Currently, a number of 3D virtual fitting rooms exist, from companies like Optitex and CLO3D. However, the current technology is plagued by two important problems, which are visualization quality as described briefly in the preceding paragraphs and the need for garment computer-aided design (CAD) patterns, as in the online retail market, the availability of CAD patterns is severely limited. Despite these problems, Fits.me, whose mannequin robots are also used for the experimental work in this paper, has a robust virtual fitting room. Instead of 3D visualization (Zhou et al., 2012), Fits.me take photos of real garment in all their available sizes on shape-changing mannequin robots to depict how the garment fits and drapes on all possible body shapes. The solution is relatively successful on the market with 25 clients worldwide, including Hugo Boss and QVC. Yet, there exists problems such as expenses required for photography process and also functionality is limited.

A couple of online fitting service providers have reached commercially successful virtual fitting rooms (Chang et al., 2013; CLO Virtual Fashion Inc., 2015; Optitex, 2015), which, in the best case, are considerably limited despite the significant advancements and advantages brought by mesh-based approaches (Liu et al., 2013). However, in order to accomplish an algorithm suitable for the whole spectrum of the market, this paper, which enables a realistic 3D virtual fitting room capable of visualization of the cloths on the digi-tailor mannequin robots adjusted based on the gathered information of them, is proposed. In fact, the scope of the paper is devised in a way that the widest possible range of all the sizes and shapes for both of the genders could be considered. First, the customer is scanned, which can be done using a variety of devices, such as depth cameras (Hauswiesner et al., 2011; Tong et al., 2012; Shotton et al., 2013). Then, the scanning instance will be analyzed to automatically calculate the main body measurements needed for classifying it based on gender. Next, another set of measurements used for classifying the person having been scanned based on the size will be estimated.

Further processing of the data obtained in the foregoing steps helps determine if the visual data provided by the laser scanner belongs to classes of male or female and small, medium, or large. Afterward, if applicable, one of the prepared size templates for the associated mannequin robots will be used, or, if not, a new set of positions of the bioengineered actuators for producing the new body shape, according to the important body measurements, including bust, waist and hip girths, will be calculated though interpolating between the already-existing size-dictionary templates, which will be selected based on the closest Euclidean distance of the template sizes from the desired body sizes. It is worth noticing that the interpolation strategy suggested and implemented throughout the paper is general enough to be applied to a wide range of biotechnological systems, such as bionic and biomimetic robots utilized for producing shapes and behaviors similar to humans.

The mannequin robots utilized as case-study for the validation of the methodology proposed and implemented throughout the paper take advantage of bio-aware and bio-inspired actuators (Anton et al., 2004; Punning et al., 2004), which constitute a substantial element required for the design and development of the systems resulted from the enhancements brought about by the physical and chemical areas of research addressing the virtue of bionics. In fact, developing, testing, and analyzing such robotic systems provide an invaluable infrastructure for improving the properties of biomimetic robots through imitating bioengineered, human-like characteristics as realistically as possible, which, among others, includes shape, locomotion, and behavior. The foregoing subject, although still in preliminary stages, is of paramount importance when it comes to improving biomimetic systems, aiming at mimicking natural creatures and processes for the purpose of understanding and modeling their specifications, which also leads to more robust construction of cybernetics prostheses, such as implants and bio-hybrid systems, relying on the biotechnological analytical information in the design stage.

The remainder of the paper is organized as follows. First, the general objectives and the economic feasibility of the virtual try-on process are described through briefly reviewing the existing literature. Then, the theoretical and technical aspects of the classification algorithm are elaborated, along with the description of the details of the steps taken toward gathering the 3D scanning database required for developing and testing it, as well as a couple of modules required for the automatic activation of the mannequin robots, which include the circuitry used for the simultaneous connection of several mannequin robots of different genders and sizes to the classification and interpolation MATLAB scripts. Afterward, the mathematical explanation of the interpolation process utilized for adjustment of the positions of the biologically inspired actuators of the mannequin robots while producing particular body sizes and shapes is presented, which is followed by a concise discussion about the practical importance and reliability of the results. Finally, the paper concludes upon pointing out the steps remaining till the completion of the whole online fitting utility, and summarizing the main points dealt with.

## 2 Literature Review of the State-of-the-Art Techniques

Clothing is a, roughly, €1 trillion market worldwide, but only 10% of it is sold online whereas the share of online sales in other goods such as books is about 50%. The weakness of online apparel sector is largely due to lack of fitting rooms. In what follows, a set of statistics and projections that are extracted according to the publicly accessible online information, are concisely reviewed, which can clearly demonstrate the strong potential of the idea of online fitting being investigated in this paper.

The statistics point out that the global manufacturing market has grown 6.9% in 2011, and presented a value of $774.4 billion, where the predicted corresponding figure for 2016 is $1,063.4 billion, showing an increase by 37.3% with respect to that of 2011 (Research and Markets, 2013a,b). It should be noted that apparel has the biggest share of the global manufacturing industry, i.e., 63.1% (671 billion) (Research and Markets, 2013a,b). At 4% projected growth for overall apparel industry, manufacturing will be valued at $755 billion in 2020 (Research and Markets, 2013a,b). Besides, customized clothing market is expected to be worth $34 billion by 2020, which will be 4.5–5% of apparel manufacturing market (Research and Markets, 2013a,b), also corresponding to the 2013 Report by European Commission Business Innovation Observatory, which states that customized clothing market is expected to be worth €27.2 billion by 2020 (European Commission, 2013).

An ideal realistic virtual fitting process, as the name says, should be aimed at representing the reality as closely as possible. In other words, the fundamental goal for the whole procedure of developing and designing the online try-on of clothes is to most realistically predict how the costumer will look like after actually wearing it, according to the present virtual information. Obviously, failure to do so, i.e., the case where the predicted visual representation differs significantly form the reality, would incur considerable waste of money and time. The latter problem is, in the most cases, caused by the incompetence of the 3D modeling and visualization algorithm in mimicking the reality correctly according to physical laws and mathematical relations. Besides, the foregoing algorithm should be accurate and precise enough to be able to adjust the output visual model appropriately according to the details of the garment, as well as the associated body shape and sizes, even when it comes to the most negligible features.

An issue to be seriously considered is to tailor the final service to the needs and desires of each customer, instead of basic mass customization. From literary point of view, mass customization is a hybrid of mass production and customization. Pine (1993) defines mass customization as the mass production of individually customized goods and services. The prerequisite of implementing mass customization is the application of advanced technology, such as the flexible manufacturing system, computer-integrated manufacturing, computer-aided design, and advanced computer technology. According to Global Cosmetic Industry (GCI) Magazine, Allured Business Media (2013), “Tailored, personalized, bespoke – whatever the word, it is clear that solutions targeted to the individual have become increasing important. In apparel, we have seen a revival of bespoke tailoring, which has dovetailed with the growth of men’s wear.” As far as the authors are concerned, the products of the whole package, although general enough to be used in mass production, will be adjustable according to the gender-, shape-, and size-specific characteristics of the customers, so that both the expectations of the shoppers in terms of flexibility and accuracy of the service, and the financial efficiency requirements would be satisfied simultaneously.

Besides paying due attention to optimizing design and manufacturing, a performance-driven attitude toward the functional processes should be considered, which searches for approaches that try to deliver higher quality products at significantly lower costs. Thus, the business model and collection of tactical method processes will be complying with the definition of lean manufacturing, meaning that we will emphasize eliminating non-value added activities (waste) while delivering quality products on time at the lowest possible cost with greater efficiency.

The foreseen system should provide fit information and recommendations for upper body garments, dresses, and trousers, including jeans. A visual representation of the shopper while prospectively wearing the cloth will be created, which requires the shoppers to be scanned. Thus, they will be able to virtually try-on various clothes and accessories, giving the chance to preview products without trying them on physically. The prototype should also be especially suitable for retailers with shallow stock policies.

In fact, one of the main goals of the paper is to come up with appropriate classification and interpolation calculation processes, which could satisfy the requirements of an appropriate methodology for adjustment of the aforementioned physical mannequin robots utilized by garment design and fashion-related industries. In order to do so, the following main steps will be taken into account:

- The garment is tried on physical mannequin robots, and an initial size dictionary is created upon producing a couple of sizes and shapes scattered in indifferent ranges. The number of the required templates in the size dictionary for this part is determined according to the experiences and the results of consultations with the experts having spent a considerable amount of time on such an issue so far;
- The resulting data obtained by scanning the mannequin robots is incorporated into the training algorithm to allow the garment shape and fabric properties make effect on the decision-making process;
- Through interpolating between different shapes in the initial size dictionary, other shapes and sizes are calculated as needed;
- Finally, putting the garments on the mannequin robots while producing the calculated sizes according to the interpolated actuator positions, and photo-shooting them creates the corresponding representation of the body while wearing the cloth.

## 3 Methodology of the Proposed Technique

In this section, the details of the stages required for classification of the scanning instances based on gender and size, as well as the characteristics of the database used for improving and testing it, are explained.

### 3.1 Scanning process

For the aim of the paper, a substantial database of 3D human body scanning data was required, for which end, scheduled scanning processes were performed on a variety of persons. The database considered for testing the algorithm contains 312 instances, including 140 males and 172 females, where in order to ensure the applicability of the proposed interpolation algorithm to all the possible categories of size, nearly equal number of small, medium, and large samples are taken into account for each gender. The results of implementation of the algorithm on the database demonstrate consistent accuracy for different genders and sizes, which proves its reliability and robustness. Similarly, due to the fact that the general format of the body, as well a couple of specific body distances and girths, might vary from age to age, various ranges are included in the database.

### 3.2 Classification and measurement ratios

The classification algorithm proposed and utilized in this paper consists of two layers, where one of the layers classifies the scanning instances into classes of male and female, and the second determines to which of the three classes of size, i.e., small, medium, and large, each person having been scanned belongs. The mathematical framework for the classification process is developed on the basis of maximum-likelihood function. The ratio of bust girth to the under-bust circumference differs significantly between the two genders, and therefore, is considered as the criterion for the classification process according to gender. On the other hand, for the classification according to size, separate gender-specific combinations of thresholds on the cross-shoulder and maximum belly circumference measurements are taken into account as the criteria.

It should be noted that for the classification based on size, initially, just single measurements had been considered as criteria. However, the latter attitude led to a significant amount of inaccuracies. Therefore, in order to reduce the number misclassification instances, all the scanning data were tested with the classifier, and the possible reasons causing the inaccuracies were investigated. For example, one of the criteria being used in the classification process, namely, the ratio of chest girth with respect to the body height, was questioned for it was not the most solid one. Therefore, we tried to search for more suitable criteria, such as the ratio of the corresponding profile measurements, and replace the previous ones by them. Besides, some of the criteria on the measurements, even if properly selected, had not been used along with the most appropriate thresholds, i.e., they were able to help classify the data with an acceptable level of accuracy, however, still needed adjustment on the associated thresholds in order for them to further reduce the frequency of misclassification instances.

Closer investigation of the measurement values considered so far for the classification algorithm based on gender led us to the conclusion that there still exist possibility of improving the performance by taking into account revised measurements. As aforementioned, in the latest version of the classification algorithm, we have considered the ratio of bust girth to under-bust circumference.

Besides, for the classification procedure according to size, we were using the cross shoulder and belly circumference values, where being assigned to a class necessitates simultaneous satisfaction of separate boundary conditions on the measurements being considered as criteria. Again, they are quite distinctive.

### 3.3 Connection of MATLAB with multiple mannequin robots

One other step is enabling MATLAB to recognize, communicate with, and send commands to several mannequin robots at the same time after the classification processes. In other words, in order to facilitate and accelerate the visual representation process, MATLAB will communicate with several mannequin robots simultaneously, and activate the one chosen in the classification process automatically in order to produce the desired body shape. The simultaneous connections are handled by a USB hub, which identifies each mannequin robot corresponding to different genders and sizes, and delivers the required actuator position information to the one commanded by the MATLAB script output, which means that after scanning each person and obtaining the automatically calculated measurements, the classification and mannequin robot activation processes work instantly, and the body sizes mimicking the body of the person having been scanned are produced by the mannequin robot in real-time.

## 4 Experimental Results of Interpolation of the Sizes

As the last module required for the purpose of the paper, one has to devise an algorithm for automatic estimation of the information required for the size dictionary of the mannequin robots. More clearly, so far, the size dictionaries have been developed in a completely manual manner. In fact, in the foregoing manual process, each time, for making the mannequin robot mimic a particular body shape, which corresponds to a particular set of the body sizes, one operator changes the position of the actuators in the software, and another one measures the important sizes manually with help of a tape meter.

First of all, there is no clear correlation between the movements in the layers and the important measurements, i.e., bust, waist, and hip girth. More clearly, from mechanical point of view, the blades can be pushed outward according to the movement of the corresponding actuator, but there is not any means for making the move inward in the case of intending to lower the size. As a result, elastic covers have been used for long time to both make the blades move inward according to the movement of the actuators, and achieve an appropriate presentation of the mannequin robot, where also the cloth can be put on it for photo-shooting purposes. As a result of the elastic nature of the covers, there is no specific relation, or even correlation, between the actuator positions and mannequin robot sizes. So, it is understandable that even after careful adjustment of the important body sizes, while trying to deal with the other ones, the previous measurements will be ruined.

The final result will be terribly limited. For example, assume that we create even 100 size templates for a mannequin robot (where the actual number is normally much lower than that). However, for a typical mannequin robot, in practice, it is possible to create billions of body sizes and shapes. Although not all the sizes are not necessarily required, but simple comparison of the two values above shows how the potential flexibility of the mannequin robots to produce any desired size, even with accuracy of less than a millimeter, has been ignored all the time, only due to the lack of a procedure for automatic creation of the size templates.

However, when calculating the actuator positions required for producing any new body shape and sizes, each of the important body measurements falls between the ones corresponding to two of the consecutive size templates, for each of which the associated actuator positions have been previously recorded in the initial database. Therefore, through calculating the distance between the desired size and each of the consecutive template sizes, and subsequently, the ratio between them, the obtained ratio can be used to estimate how the required actuator position should be adjusted.

However, for each particular bust, waist, or hip size, there are numerous combinations with the measurements of other important body sizes from different ranges. Therefore, given the fact that, as aforementioned, the changes of the sizes in each region are not independent from the changes of the sizes in the other layers, one should try to determine the size templates that, apart from resembling the same initial and final interpolation values for the corresponding measurement layer, present other values as close to that of the desired body shape as possible. For example, when interpolating the actuator positions required for producing a particular bust girth size, *D_b_*, for a specific body shape, as initial and final values, among the size templates in the forms $\left(P_{b},\;M_{w_{j}},\;M_{h_{j}}\right)$ and $\left(N_{b},\;M_{w_{j}},\;M_{h_{j}}\right)$, respectively, the two should be taken into consideration that not only present bust values previous and next to the corresponding bust value, i.e., *P_b_* and *N_b_*, respectively, but also resemble the waist and hip girth sizes that are closest, in the sense of Euclidean distance, to that of the desired shape.

More precisely, considering a sample small female mannequin robot as an example, the mathematical relation needed for mapping the calculated ratio to the distance between the actuator positions corresponding to the sizes included in the aforementioned size templates and obtaining actuator positions located in points maintaining the same ratio between them can be formulated as follows:

(1) $$\begin{array}{c} d_{b_{i}}=w_{b_{p}}p_{b_{i}}+w_{b_{n}}n_{b_{i}},\\ i=51,52,\ldots ,56,\;\text{or}\;i=61,62,\ldots ,66, \end{array}$$

(2) $$\begin{array}{c} d_{w_{i}}=w_{w_{p}}p_{w_{i}}+w_{w_{n}}n_{w_{i}},\\ i=31,32,\ldots ,36,\;\text{or}\;i=41,42,\ldots ,46, \end{array}$$

(3) $$\begin{array}{c} d_{h_{i}}=w_{h_{p}}p_{h_{i}}+w_{h_{n}}n_{h_{i}},\\ i=11,12,\ldots ,16,\;\text{or}\;i=21,22,\ldots ,26, \end{array}$$

where

(4) $$\begin{array}{cc} w_{b_{p}}=\frac{N_{b}-D_{b}}{N_{b}-P_{b}}, & w_{b_{n}}=\frac{D_{b}-P_{b}}{N_{b}-P_{b}},\\ w_{w_{p}}=\frac{N_{w}-D_{w}}{N_{w}-P_{w}}, & w_{w_{n}}=\frac{D_{w}-P_{w}}{N_{w}-P_{w}},\\ w_{h_{p}}=\frac{N_{h}-D_{h}}{N_{h}-P_{h}}, & w_{h_{n}}=\frac{D_{h}-P_{h}}{N_{h}-P_{h}}, \end{array}$$

which leads to

(5) $$\begin{array}{c} d_{b_{i}}=p_{b_{i}}+\left(n_{b_{i}}-p_{b_{i}}\right)\frac{D_{b}-P_{b}}{N_{b}-P_{b}},\\ i=51,52,\ldots ,56,\;\text{or}\;i=61,62,\ldots ,66,\\ d_{w_{i}}=p_{w_{i}}+\left(n_{w_{i}}-p_{w_{i}}\right)\frac{D_{w}-P_{w}}{N_{w}-P_{w}},\\ i=31,32,\ldots ,36,\;\text{or}\;i=41,42,\ldots ,46,\\ d_{h_{i}}=p_{h_{i}}+\left(n_{h_{i}}-p_{h_{i}}\right)\frac{D_{h}-P_{h}}{N_{h}-P_{h}},\\ i=11,12,\ldots ,16,\;\text{or}\;i=21,22,\ldots ,26, \end{array}$$

for the actuators located in the layers affecting the bust, waist, and hip girths, respectively, where *i* is a dummy variable, $d_{b_{i}}$, $d_{w_{i}}$, and $d_{h_{i}}$ stand for the desired position of the actuators of the activated mannequin robot, respectively. It should be mentioned that in the above formulation, $p_{b_{i}}$, $p_{w_{i}}$, and $p_{h_{i}}$ denote the actuator positions in the size template containing the initial interpolation values, where $n_{b_{i}}$, $n_{w_{i}}$, and $n_{h_{i}}$ represent the actuator positions in the size template resembling the final interpolation values, respectively, for the actuators affecting the bust, waist, and hip girth sizes. Besides, *D_b_*, *D_w_*, and *D_h_* stand, respectively, for the desired values of bust, waist, and hip girths. Moreover, the sets (*P_b_*, *P_w_*, *P_h_*) and (*N_b_*, *N_w_*, *N_h_*) denote the bust, girth, and hip girth sizes in the initial and final interpolation size templates, respectively, which are the solutions to the following optimization problems:

(6) $$\begin{array}{c} \min\left(\sqrt{{\left(D_{w}-M_{w_{j}}\right)}^{2}+{\left(D_{h}-M_{h_{j}}\right)}^{2}}\right)\\ \text{over}\;j=1,\ldots ,m,\\ \text{Subject to}\;\exists j:\;\left(P_{b},M_{w_{j}},M_{h_{j}}\right)\in M, \end{array}$$

(7) $$\begin{array}{c} \min\left(\sqrt{{\left(D_{b}-M_{b_{j}}\right)}^{2}+{\left(D_{h}-M_{h_{j}}\right)}^{2}}\right)\\ \text{over}\;j=1,\ldots ,m,\\ \text{Subject to}\;\exists j:\;\left(M_{b_{j}},P_{w},M_{h_{j}}\right)\in M, \end{array}$$

(8) $$\begin{array}{c} \min\left(\sqrt{{\left(D_{b}-M_{b_{j}}\right)}^{2}+{\left(D_{w}-M_{w_{j}}\right)}^{2}}\right)\\ \text{over}\;j=1,\ldots ,m,\\ \text{Subject to}\;\exists j:\;\left(M_{b_{j}},M_{w_{j}},P_{h}\right)\in M, \end{array}$$

and

(9) $$\begin{array}{c} \min\left(\sqrt{{\left(D_{w}-M_{w_{j}}\right)}^{2}+{\left(D_{h}-M_{h_{j}}\right)}^{2}}\right)\\ \text{over}\;j=1,\ldots ,m,\\ \text{Subject to}\;\exists j:\;\left(N_{b},M_{w_{j}},M_{h_{j}}\right)\in M, \end{array}$$

(10) $$\begin{array}{c} \min\left(\sqrt{{\left(D_{b}-M_{b_{j}}\right)}^{2}+{\left(D_{h}-M_{h_{j}}\right)}^{2}}\right)\\ \text{over}\;j=1,\ldots ,m,\\ \text{Subject to}\;\exists j:\;\left(M_{b_{j}},N_{w},M_{h_{j}}\right)\in M, \end{array}$$

(11) $$\begin{array}{c} \min\left(\sqrt{{\left(D_{b}-M_{b_{j}}\right)}^{2}+{\left(D_{w}-M_{w_{j}}\right)}^{2}}\right)\\ \text{over}\;j=1,\ldots ,m,\\ \text{Subject to}\;\exists j:\;\left(M_{b_{j}},M_{w_{j}},N_{h}\right)\in M, \end{array}$$

respectively, where *j* = 1, …, *m* is a dummy variable, and

(12) $$\begin{array}{l} M=\left\{M_{j}|j=1,\ldots ,m\right\}\\ =\left\{\left(M_{b_{j}},M_{w_{j}},M_{h_{j}}\right)|j=1,\ldots ,m\right\} \end{array}$$

is the global set containing all the size templates. It is worth noticing that, in order to make sure that the size template values immediately smaller/greater than the desired sizes will be considered as the initial and final interpolation values (*P_b_*, *P_w_*, *P_h_*) and (*N_b_*, *N_w_*, *N_h_*) sets are considered such that

(13) $$\begin{array}{c} P_{b}<D_{b}<N_{b},\\ ∄M_{j}\in M:\;P_{b}<M_{b_{j}}<D_{b},\\ ∄M_{j}\in M:\;D_{b}<M_{b_{j}}<N_{b},\\ P_{w}<D_{w}<N_{w},\\ ∄M_{j}\in M:\;P_{w}<M_{w_{j}}<D_{w},\\ ∄M_{j}\in M:\;D_{w}<M_{w_{j}}<N_{w},\\ P_{h}<D_{h}<N_{h},\\ ∄M_{j}\in M:\;P_{h}<M_{h_{j}}<D_{h},\\ ∄M_{j}\in M:\;D_{h}<M_{b_{h}}<N_{h}, \end{array}$$

where if one or more of the desired sizes matches one of the size-dictionary values exactly, the interpolation process for that particular size will be ignored, and just the size template determined according to the corresponding optimization problem from the equations (6) through (11) will be directly used for adjusting the positions of the associated actuators. Besides, if one of the measurements is not within the limits determined by the mechanical constraints, in order to avoid damaging the actuators and blades, the closest value falling inside the allowed ranges, i.e., the minimum or maximum, depending on if the value is lower than the minimum or higher than the maximum, respectively, will be considered instead.

Performing the described interpolation, although roughly correct, does not guarantee that the body sizes will also change according to a simple proportional relationship, but in most of the cases, due to a variety of issues, including the aforementioned elastic nature of the cover, etc., the proportions of the variations of the body sizes between templates is always negligibly different from that of the changes of the actuator positions. However, our preliminary experimentations showed that the foregoing inaccuracies are tolerable, at least, compared to the level of inaccuracy of the process followed so far.

**Figure 1:**
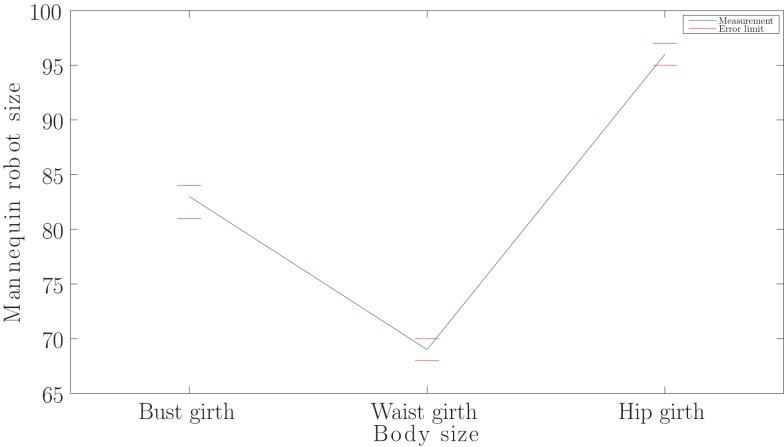
**Illustration of three of the important body sizes, namely, bust, waist, and hip girths, of a small female sample, used in the classification and interpolation processes, along with the mannequin sizes resulted from the interpolation of the size dictionaries, where the maximum inaccuracy limits of the foregoing procedure are shown separately**.

**Figure 2:**
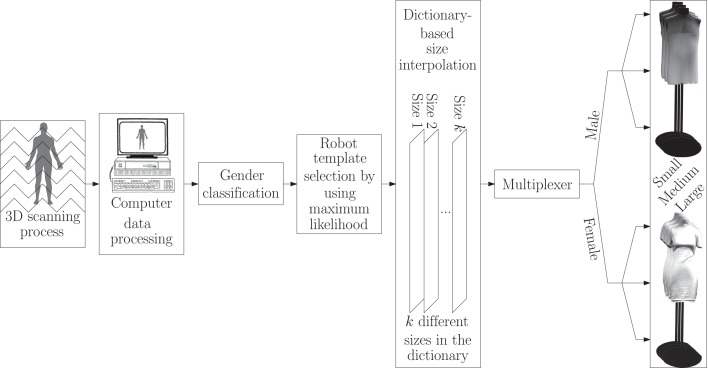
**A flowchart showing the whole classification and automatic adjustment processes in a glance**.

For the purpose of calculating the tolerance of the size-dictionary interpolation algorithm and visually illustrating the maximum differences between the measurements provided by the actuator positions calculated by the latter process and the actual desired values, 312 scanning instances were tested. The resulting accuracy plot demonstrates the desired measurements of one of the samples along with the intervals within which the ones resulting from the interpolation algorithm might fall, i.e., the maximum tolerances of the error between the foregoing sets of measurements according to all the investigated sets of scanning instances are shown. The latter tolerances can be shown as follows:

(14) $$\begin{array}{ccc} bg_{m} & \in \left[bg_{d}-2,bg_{d}+1\right], & \\ wg_{m} & \in \left[wg_{d}-1,wg_{d}+1\right],\\ hg_{m} & \in \left[hg_{d}-1,hg_{d}+1\right], \end{array}$$

where bg_d_, wg_d_, and hg_d_ denote the desired bust, waist, and hip girths, respectively, and bg_m_, wg_m_, and hg_m_ stand for the bust, waist, and hip girth measurements resulted from the size-dictionary interpolation process, respectively. Figure 1 illustrates the foregoing accuracy plot. Besides, a summary of the processes corresponding to classification, size interpolation, and automatic activation of the mannequin robots is schematically illustrated in Figure 2.

## 5 Discussion

The novelty of the approach proposed in the paper arises from the obviation of the necessity of manually preparing body templates, which has been considered only solution to the problem of imitating the shape in most of the similar studies previously reported in the literature (Abels and Kruusmaa, 2013). In fact, the current algorithm alleviates the process as a whole, both in the sense of time-efficiency and accuracy. Regarding the former, with the conventional process, preparing the templates was extremely time consuming, and took around a full working week for two operators to prepare the basic dictionary for each mannequin robot. Besides, the templates had to contain specific measurements, which needed to be achieved through trial and error. However, with the proposed method, substantial number of templates can be obtained within a few minutes, which do not have to follow any specific pattern, and can be randomly generated. The only requirement that might prove helpful is that the template sizes be fairly scattered throughout the whole possible range, which further increases the resulting accuracy and flexibility, and ensures the feasibility of mimicking body sizes and shapes from the widest possible range of measurements from various segments.

As for the accuracy, since the manual templates were difficult to prepare, their number was limited to a certain amount, where, in the best case, i.e., with the highest possible number of templates, and the most recent version of the mannequin robot, the sizes and girths corresponding to two consecutive templates used to differ at least 4 cm, meaning that the least amount of allowed inaccuracy was 2 cm. However, the current algorithm, in case of most of the important body sizes, maximally demonstrates 1 cm of inaccuracy, which has proved desirable from the point of view of virtual fitting room providers, online garment retailers, and fashion designers. It is worth noticing that one of the possible drawbacks that the proposed methodology might entail is that it demands the existence of a 3D laser scanner, which is not necessarily available to all the potential target users from the aforementioned list.

## 6 Conclusion

This research work presented the classification and size-dictionary interpolation modules of a realistic virtual fitting room, along with providing the chance to activate the mannequin robot chosen by the classification algorithm in order to produce the corresponding body shape and sizes instantly, where several mannequin robots representing different genders and sizes could be simultaneously connected to the same computer. The classification stage included separate elements dealing with gender and size. In the foregoing modules, a set of distinctive body measurements provided by the automatic measurement wizard of the 3D laser scanner were taken into consideration. The choice on the measurements was based on the level of the difference between them when compared according to the scanning instances form different genders and sizes, and the maximum-likelihood function was considered as the criterion. The size-dictionary interpolation module was aimed at estimating the proper positions of the biologically inspired actuators required for producing certain body shapes and sizes according to that of the ones already existing in the database. The latter algorithm was based on making a linear map between the size templates and the associated positions of the bioengineered actuators, where separate interpolations were performed for maintaining the same ratio as that of the sizes proportions between the actuator positions. For choosing the closest possible combinations of the body sizes among the templates as the initial and final interpolation points, the Euclidean distance of the template size vectors from the desires sizes was taken into consideration as the objective of the optimization algorithm. Finally, the results were visually represented, and the competence of the proposed classification and size-dictionary interpolation package was verified, which is well-applicable to all sorts of biotechnological shape-changing robots meant to mimic living-being-like shape or behavior, including among others, the ones aimed at bionics purposes.

## Conflict of Interest Statement

The authors declare that the research was conducted in the absence of any commercial or financial relationships that could be construed as a potential conflict of interest.
